# Illogical and Unethical Scientific Sanctions Against Iranian Authors

**Published:** 2013

**Authors:** Mehran Zarghami

**Affiliations:** 1 Professor, Psychiatry and Behavioral Sciences Research Center AND Department of Psychiatry, School of Medicine, Mazandaran University of Medical Sciences, Mazandaran, Sari, Iran.

## Abstract

Restriction of free submission of Iranian manuscripts is not a new matter. However, the recent political decision of the USA Office of Foreign Assets Control of the Department of the Treasury saying who can and who cannot publish scientific papers in journals is completely against free distribution of data in scholarly and scientific communities and scientific journals and publications which requires discussions and challenges with the statutory authorities. Publishers’ mission requires bringing suit against this illogical and unethical decision. Science should not be involved in the political game of sanctions. Policies of governments and the origins of the manuscript, including the race, ethnicity, nationality, religion, and political belief of the authors should not affect the editorial decisions of scientific publications.

**Declaration of interest:** None.

“We are sorry for the delay in getting back to you with a decision about your submitted manuscript.

Because you and your authors are from an Iranian government-supported institution, we cannot consider your manuscript for publication in the Journal of Clinical Psychopharmacology. This policy is a United States policy that is now in effect, and therefore, we are required to withdraw your paper from consideration.” This is what Barbara Kern, the Managing Editor of the Journal of Clinical Psychopharmacology, Tufts University School of Medicine, Boston, said in response to an Iranian resident of psychiatry who had submitted her manuscript about treatment of schizophrenia, not about nuclear bombs, terrorism, chemical weapons, etc...

Restriction of free dissemination of information and submission of Iranian manuscripts is not a new matter. This problem arises from USA federal rules in 2004 that undermine a codified policy order “to protect the constitutional rights of Americans to educate themselves about the world by communicating with peoples of other countries in a variety of ways, such as by sharing information and ideas with persons around the world, traveling abroad, and engaging in educational, cultural, and other exchanges with persons from around the world” (2). As mentioned by Eliot Marshal, ScienceInsider writer, it has put in place much harsher restrictions on communications from Iran since a decade ago (3). Publishers brought suit against the USA government at that time and succeed in their effort (4). The regulations changed; preserved the “informational materials” as exception and made a general certification for publishing activities, particularly peer reviewed, author, and editorial activities in 2007 (2). However, over recent years, the United Nations, the European Union, and recently the United States of America have proposed more and more limiting trade sanction regulations in relation to Iran, and some of these restrictions impact publications (2). For example, the Elsevier Company had applied "more specific sanctions … over the past year or two" as a result of UN advises (3).

Regarding the “fast-rejection” by the Journal of Clinical Psychopharmacology we inquired about this policy from another American journal. According to their response in an e-mail it seems that they are obliged to reject the papers written by authors affiliated to the government of Iran. They claim that these sanctions aimed to persuade the Iranian government to give up “nuclear technology”, and are a broadened decision from previous rules issued by the enforcement agency, the USA Office of Foreign Assets Control (OFAC) of the Department of the Treasury (3). Under this policy reflecting the USA Congress law even companies not based in the United States must prevent their American personnel from interacting with these authors (3). Moreover, the American Journal of Cardiology and the Journal of Allergy and Clinical Immunology (JACI), which are published by Elsevier, the Journal of Ocular Pharmacology and Therapeutics (published in the US by Marie Ann Liebert), and the Australian journal Ophthalmic Epidemiology (owned by Informa Healthcare in the UK), Dove Medical Press (an open access online publisher whose headquarters are in the UK and editorial office is in New Zealand) have adopted a similar strategy (5). 

Despite the illogical and unethical political decision of OFAC, we assume authors affiliated with academic and research institutes and manuscripts originating from hospital and clinical settings are not involved in this political game. “The focus of our sanctions is the Iranian regime and its support for terrorism and its illicit nuclear programme. Our sanctions do not target academic or informational materials” Saied John Sullivan of the US Treasury stated to the British Medical Journal (BMJ) (5). As mentioned by an Iranian author in an open letter, “at a time when several countries, even the USA, despite important differences of opinion are trying to reduce tensions with Iran (e.g. withdrawal of sanctions on items such as computer software and hardware, mobile phones, food, and medicine), discriminatory action against scientific publishing is difficult to understand” (6).

An important matter is the “lability phobia” of the scientific journal editors who have enforced extra sanctions beyond the OFAC sanctions for Iranian academic authors. For example, Elsevier has noted the new restrictions are limited to editors who are USA nationals, have an exception for Iranian universities and health-care institutions, and are expected to affect a small number of papers (such as research departments of nuclear facilities and various oil and gas companies which are deemed to be entities of the Government of Iran). However, this publisher believes that it is necessary to make “narrowly crafted exceptions” since there is a potential risk of personal “liability” on the part of its USA editors (2, 3). Jeanette Pearce, Dove’s New Zealand based operations manager said: “We do not make this decision lightly and have consulted with the authorities both here in New Zealand and in the United Kingdom before making our decision”(5). The editor of the Journal of Allergy and Clinical Immunology (JACI) explained in an e-mail that: “Our publisher precludes taking any articles from authors based at Iranian institutions or hospitals.”(5). “Our recent communications with editors were motivated out of concern that U.S. citizens acting as editors of our journals might also be subject to personal liability” said Eliot Marshal, ScienceIncider writer (3). 

In practice, as almost all of the Iranian universities of medical sciences, and academic and research institutes are run with governmental budget, their submission should be rejected outright. On the other hand, the new law specifies that the sanctions do not apply to any academic and research institutions, or their staff or informational materials, and the USA, OFAC, has used new regulations to target certain Iranian nationals, including Iranian government employees (but not Iranian universities) that are supposed to be contracted in or assisting the development of weapons technologies or supporting the government-owned energy industry. However, it seems that fear of personal penalty has made scientific journal editors more conservative (2, 5). Although the Elsevier Company states that free flow of ideas and information is a principle for science and for society, the statements of Mark Seeley the chief lawyer, regarding the misunderstanding of some US editors on the publisher’s instructions, confirms the “lability phobia” and conservativeness. “The problem is over and the exact definition of ‘government employees’ other than academics and practitioners is not clear”: Seeley said (5). Furthermore, he stated that the Elsevier Company will try to dispel this ambiguity, but has not done so yet (5). 

On the other hand, some other American and British journals, including the Journal of American Medical Association (JAMA), the New England Journal of Medicine, and the British Medical Journal (BMJ) continue to publish Iranian submissions (5). The Lancet (which is published by Elsevier) is currently working to strengthen its links with Iranian medical and public health scientists (5). Several other scientific organizations said that OFAC tangled with scientific journals; they did not see a need to alter the way they manage manuscripts at present (3). “I certainly do not condone what Iran or its government says or does (or for that matter any country that has less than positive relations with the US). “Please leave this kind of politics out of science” said the Journal of Pharmacological and Toxicological Methods on behalf of … (deleted by them to avoid abuse) (7). In this regard, political scientists believe that the impact of sanctions on their stated objectives is very low (8).

I believe that free dissemination of information in scholarly and scientific communities, and scientific journals and publications requires discussions with the statutory authorities regarding revisions of regulations. The Iranian Journal of Psychiatry and Behavioral Sciences endorses the following recommended clause of The Committee on Publication Ethics (COPE), which is drawn from the text developed by World Association of Medical Editors (WAME). This clause is that:

“Editorial decisions should not be affected by the origins of the manuscript, including the nationality, ethnicity, political beliefs, race, or religion of the authors. Decisions to edit and publish should not be determined by the policies of governments or other agencies outside of the journal itself.” (9).

## Footenote

*“The ones … who listen to the statement and follow the best in it, will have good news: proclaim such to My servants. Those are the ones whom God has guided; those are prudent persons.” (The Holly Quran)(* 1 *)*
